# Deep-Learning Model of ResNet Combined with CBAM for Malignant–Benign Pulmonary Nodules Classification on Computed Tomography Images

**DOI:** 10.3390/medicina59061088

**Published:** 2023-06-05

**Authors:** Yanfei Zhang, Wei Feng, Zhiyuan Wu, Weiming Li, Lixin Tao, Xiangtong Liu, Feng Zhang, Yan Gao, Jian Huang, Xiuhua Guo

**Affiliations:** 1 Department of Epidemiology and Health Statistics, School of Public Health, Capital Medical University, Beijing 100069, China; 2 Beijing Municipal Key Laboratory of Clinical Epidemiology, Capital Medical University, Beijing 100069, China; 3 Department of Nuclear Medicine, Xuanwu Hospital Capital Medical University, Beijing 100053, China; 4 School of Mathematical Sciences, University College Cork, T12 YN60 Cork, Ireland

**Keywords:** lung cancer, CT, deep learning, radiomics, machine learning

## Abstract

*Background and Objectives*: Lung cancer remains a leading cause of cancer mortality worldwide. Accurately classifying benign pulmonary nodules and malignant ones is crucial for early diagnosis and improved patient outcomes. The purpose of this study is to explore the deep-learning model of ResNet combined with a convolutional block attention module (CBAM) for the differentiation between benign and malignant lung cancer, based on computed tomography (CT) images, morphological features, and clinical information. *Methods and materials*: In this study, 8241 CT slices containing pulmonary nodules were retrospectively included. A random sample comprising 20% (*n* = 1647) of the images was used as the test set, and the remaining data were used as the training set. ResNet combined CBAM (ResNet-CBAM) was used to establish classifiers on the basis of images, morphological features, and clinical information. Nonsubsampled dual-tree complex contourlet transform (NSDTCT) combined with SVM classifier (NSDTCT-SVM) was used as a comparative model. *Results*: The AUC and the accuracy of the CBAM-ResNet model were 0.940 and 0.867, respectively, in test set when there were only images as inputs. By combining the morphological features and clinical information, CBAM-ResNet shows better performance (AUC: 0.957, accuracy: 0.898). In comparison, a radiomic analysis using NSDTCT-SVM achieved AUC and accuracy values of 0.807 and 0.779, respectively. *Conclusions*: Our findings demonstrate that deep-learning models, combined with additional information, can enhance the classification performance of pulmonary nodules. This model can assist clinicians in accurately diagnosing pulmonary nodules in clinical practice.

## 1. Introduction

Worldwide, lung cancer remains the leading cause of death in cancer mortality. It was estimated that 2.2 million new lung cancer cases (11.6% of the total cases) and 1.8 million deaths (18% of the total cancer deaths) occurred in 2020 [1]. Early diagnosis of asymptomatic lung cancer plays a vital role in treatment planning that can significantly improve the survival rate of lung cancer patients, but only 15% of patients were diagnosed at an early stage of the pathological process, to a large extent leading to poor prognoses [2,3], where stage I and II lung cancer have a much better prognosis than stage III or IV lung cancer [4]. Computed tomography (CT) has been recognized as an effective and noninvasive early diagnostic method for nodule localization, evaluation of tumor size, morphological features analysis, benign–malignant classification, and survival prediction in patients [5]. The National Lung Screening Trial (NLST) has reported that screening with low-dose computed tomography (LDCT) scans will result in a 20% reduction in lung cancer mortalities [6,7]. Lung cancer management guidelines and data-driven models have been developed, but accurately distinguishing between benign and malignant nodules remains a challenge [7].

Deep learning has emerged in the computer vision field and become very popular in the medical imaging field [8]. Deep learning based on convolutional neural networks (CNNs) has been successfully applied in the diagnoses of various diseases, such as skin cancers [9], brain strokes [10], and lung diseases [11,12]. These CNN models have highlighted the possibility of automatically exploiting features from the images and accordingly completing the tasks of feature selection and weight tuning without requiring a complicated pipeline of image-processing and pattern-recognition steps [13,14]. Using large-scale training image data, CNN models provide a uniform framework for jointly learning the hierarchical representative features extracted directly from images and classification weights. Radiomics is an emerging technique that extracts high-dimensional quantitative image features for diagnosis and prognosis [15,16]. Texture features are the most frequently used modality, and an increasing number of studies have suggested that CT texture features have massive diagnostic value in clinical diagnosis [3,17,18]. In our previous study, we found that nonsubsampled dual-tree complex contourlet transform (NSDTCT) can describe the image in multiple scales and directions and that it can extract richer detailed information from the image. But there is no conclusion on which method can better classify pulmonary nodules. Regardless of whether using deep-learning methods or radiomic approaches, many studies have been developed for classifying and predicting the malignant risk of pulmonary nodules, but they often rely on subjective labels provided by radiologists and lack reliable reference criteria for histopathological examination [19,20,21]. Moreover, most models incorporate only image information and ignore other important information from the patients.

Herein, we have collected CT images of patients with pulmonary nodules with pathological results from multiple centers. A novel deep-learning model was proposed to classify benign pulmonary nodules and malignant ones according to CT images, morphological features, and clinical information. Radiomic models were used for comparison. This work serves as a promising diagnostic tool for the early diagnosis of lung cancer and improving patients’ survival rates.

## 2. Methods

### 2.1. Data Source

In total, 972 patients from 4 hospitals in China were recruited in this study from 2015 to 2019, and informed consent was obtained. The lung CT images of all the patients were collected in DICOM format, and together, there were 8241 slices involving lung nodules. By consulting the patient’s medical records and admission information, we continued to collect the pathological diagnosis results, demographic information, environmental and behavioral factors, and imaging signs of pulmonary nodules in the form of a questionnaire. The inclusion criteria were as follows: (1) the number of CT images containing nodules in each patient must not be less 2; (2) for malignant nodules, a pathological diagnosis or a discharge diagnosis was determined as lung cancer thanks to a doctor’s prior knowledge; for benign nodules, the patients were diagnosed with other diseases through pathological diagnosis or thanks to a doctor’s prior knowledge. The exclusion criteria were as follows: (1) patients who were treated with chemo-radiation therapy or surgery and (2) images in which the nodules were hard to segment. The checklist of subjects and images is shown in Table 1.

This study took each picture as the research object. The pictures were first split into a training set (80%) and a testing set (20%), and the positive–negative sample ratios in these sets were approximately the same as those in the complete data set. Next, the training set was used to fit and tune the models, and the testing set was used to evaluate the predictive and generalization ability of the models. The simple statistics of the training and testing sets are summarized in Table 2.

### 2.2. Image Preprocessing

**Figure 1:**
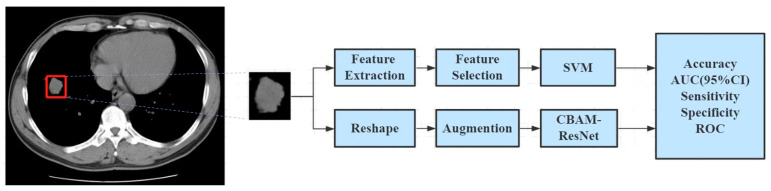
Workflow of CT image analysis to classify the pulmonary nodules.

The region of interest (ROI) was semiautomatically segmented from the whole CT image by using the region growth method, which was performed by two experienced radiologists and conducted with MATLAB 2017. An overview of the study workflow is illustrated in Figure 1.

### 2.3. Deep-Learning Algorithm

A CNN is a framework belonging to deep learning that has shown state-of-the-art performance in image segmentation and classification [22,23]. All the processes are automatically performed, and the massive weights are set and updated via a back-propagation (BP) algorithm to minimize the loss function, thus achieving the best classification accuracy. Several common CNN models, including Vgg16, GoogLeNet, ResNet, and DenseNet, perform well on different classification tasks [24,25,26,27]. We chose ResNet50 as the basic network structure for deep learning. Next, we adopted a transfer-learning strategy and data-argument technique; the pretraining weights were derived from training on the ImageNet data set, and the best parameter of the model can be obtained via fine-tuning.

**Figure 2:**
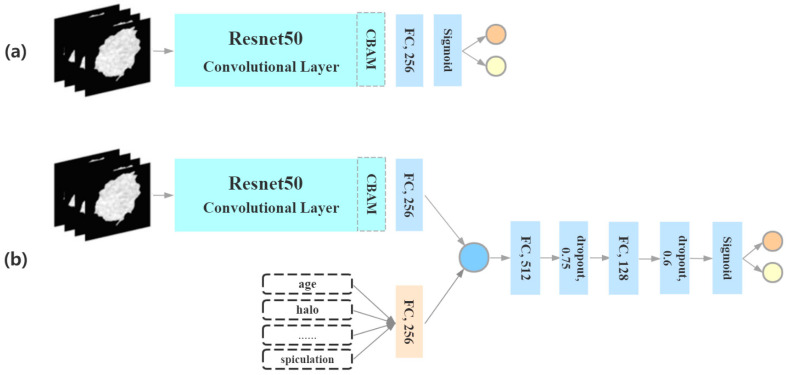
Deep-learning model structure. (**a**) CBAM-ResNet model using CT images of pulmonary nodules alone. (**b**) CBAM-ResNet model combining CT images, morphological features, and clinical information.

According to the CNN model, the greatest advantage of the ResNet framework lies in adding identity mapping that is performed by the shortcut connections, the outputs of which are added to the outputs of the stacked layers [28]. Therefore, the ResNet addresses the degradation problem and adds neither extra parameters nor computational complexity. In addition, we try to add a convolutional block attention module (CBAM) in the ResNet. CBAM is a simple and effective attention module for feedforward convolutional neural networks. Given an intermediate feature map, the CBAM infers the attention map along two independent dimensions (channel and space) in turn, and next, it multiplies the attention map and the input feature map for adaptive feature optimization [29]. It can be seamlessly integrated into any CNN architecture without factoring in the overhead of the module, and it can be trained end to end with a basic CNN. The structure of our CBAM-ResNet is shown in Figure 2.

During the model training process, the input size of the image is 64 × 64, the optimizer used is Adam, the cross entropy is employed as the loss function, the batch size is set to 128, and the maximum number of iterations is 100.

### 2.4. Radiomic Analysis

A nonsubsampled dual-tree complex contourlet transform (NSDTCT) was conducted on the ROI to obtain 96 subband images, and feature extraction was then performed on the subbands. NSDTCT has two steps. First, a dual-tree complex wavelet transform was used to decompose the original image into 2 low-frequency bands and 6 high-frequency bands of 6 directions (±15°, ±45°, ±75°); next, 2^n^ band coefficients were set on the high-frequency level by using a nonsubsampled directional filter bank. The acquired imaging features contained the texture features, including 6 texture features based on a gray histogram (average gray level, average contrast, measure of smoothness, third moment, measure of uniformity, and entropy), 14 texture features based on a gray-level co-occurrence matrix (GLCM) (energy, inertia, inverse difference of moment, entropy of GLCM, correlation, cluster of tendency, contrast, homogeneity, variance, maximum of probability, sum of mean, difference of mean, sum of entropy, and difference of entropy), and 3 texture features based on a neighborhood gray difference moment (difference entropy, coarseness contrast, and busyness). In total, 2208 features were obtained from each ROI. Feature extraction was performed by MATLAB 2017, which enables the processing and extraction of radiomic features from medical image data.

Given that some derived features could be redundant according to the classifier, we have reduced the dimensionality of the texture features. In this study, we adopted the commonly used dimensionality reduction method: least absolute shrinkage and selection operator (LASSO). LASSO is a strategy for feature selection that produces some coefficients that are exactly zero and hence exhibits simple and interpretable models. In total, 2208 texture values were calculated in feature selection. These 2208 textures were incorporated into the LASSO regression model with a penalty function, and 128 features were retained, according to the best accuracy for the following classification models. The best parameters of LASSO were selected by 10-fold cross validation.

A support vector machine (SVM) is a widely applied supervised machining-learning method that is very suitable for small sample, nonlinear, and high-dimensional pattern-recognition problems, and it has achieved good classification results on many image feature classification tasks [30,31,32]. In this research, the radial basis function (RBF) was adopted, and a 10-fold cross-validation method was used to optimize the parameters (gamma and cost).

### 2.5. Deep-Feature Visualization

Convolutional neural networks can automatically extract image features and classify them. To explore whether the model learned useful features from meaningful areas, we used two visualization methods to interpret the features extracted by the CNN.

To reveal the focus area of the model, we extracted the feature maps from the CBMA-ResNet model. Gradient-weighted class activation mapping (Grad-CAM) was used to express the importance of the features. The area with higher values on the feature map was considered to be the area that contributed more to the generated result.

The effectiveness of the learned features was shown via *t*-distributed stochastic neighborhood embedding (*t*-SNE), an unsupervised dimension-reduction algorithm for visualizing high-dimensional data. We utilized *t*-SNE to reduce the dimension of global features from 256 to 2.

### 2.6. Statistical Analysis

The statistical descriptions of clinical information and the morphological features are presented as the mean and the standard deviation (SD) or percentage, respectively; R 4.0.3 software was used to perform the χ2 test or *t*-test for the basic clinical data of patients and the morphological data of images. The difference was statistically significant at *p* < 0.05. Fill in missing values by using the random forest method on the morphological features. To evaluate the classification performance on the training set and the testing set, three indexes (accuracy, sensitivity, and specificity) were calculated. The 95% confidence interval (95% CI) of the area under the curve (AUC) was also calculated on the basis of the results from a binomial exact test. Curves from the receiver operating characteristics (ROC) were plotted to visually compare the differences between the models.

## 3. Results

### 3.1. Clinical and Morphological Characteristics

In total, 972 patients were involved in this study: 612 in the malignant group (malignant pulmonary nodule patients) and 360 in the benign group (benign pulmonary nodule patients). The male proportion comprised 62.4% of the malignant group and 61.4% of the benign group. The mean age was 61.9 in the malignant group and 54.8 in the benign group. Moreover, 25.3% of the benign cases smoked, and 46.3% of the malignant cases smoked. The statistical difference test showed that there was no significant difference in gender between the two groups, but there was a significant difference in age and smoking. The morphological feature distribution of images is shown in Table 3. According to the results, all eight features had a significant between-group difference of *p* < 0.001.

### 3.2. Performance of Classification

In this study, we combined the ResNet and CBAM (CBAM-ResNet), a novel deep-learning algorithm to classify malignant and benign lung nodules. We constructed three deep-learning models: a CBAM-ResNet model built with images as inputs (CBAM-ResNet1), a CBAM-ResNet model with images and morphological features as inputs (CBAM-ResNet2), and a CBAM-ResNet with age and smoking data from CBAM-ResNet2 (CBAM-ResNet3). Among them, eight morphological features (diameter, halo, lobulation, vacuole, spiculation, calcification, cavity, and pleural retraction) were incorporated into the model. In CBAM-ResNet3, the factors of age and smoking were transformed into categorical variables (age was divided into three groups, namely ≤50, 50 to 65, and >65 years old, and smoking was divided into smokers and nonsmokers). The NSDTCT texture feature combined with the SVM classifier (NSDTCT-SVM) was used as a comparison model. Similarly, we established three models: NSDTCT-SVM1 (using only images as inputs), NSDTCT-SVM2 (combining images and morphological features as inputs), NSDTCT-SVM3 (adding age and smoking based on NSDTCT-SVM2).

**Figure 3:**
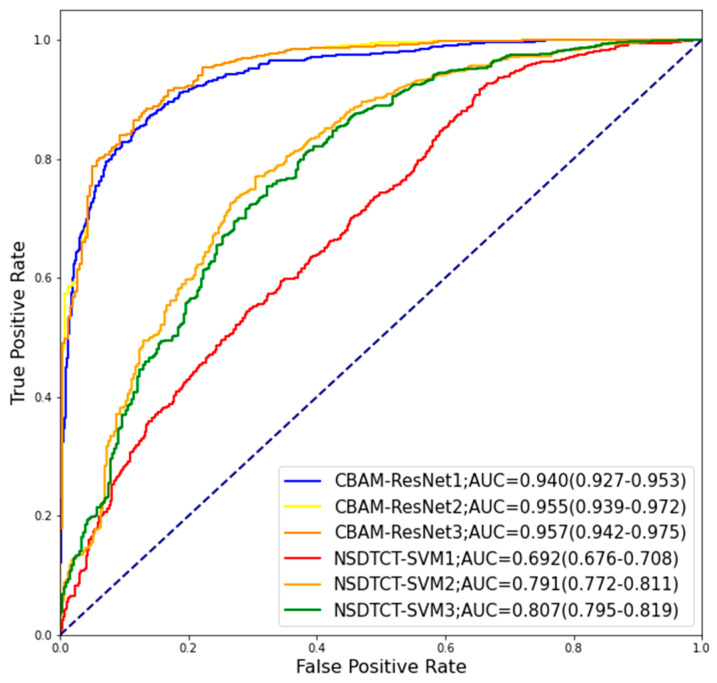
The ROC curves of four models on the test set.

The results of the six models in the test set are shown in Table 4. The ROC curves of classification models were plotted (Figure 3). In the test set, the CBAM-ResNet3 model achieved the best accuracy. The deep-learning model CBAM-ResNet3 performed better than the conventional radiomic method. By combining it with morphological information and clinical information, the performance of the model can be further improved.

### 3.3. Visualization of Deep Features

**Figure 4:**
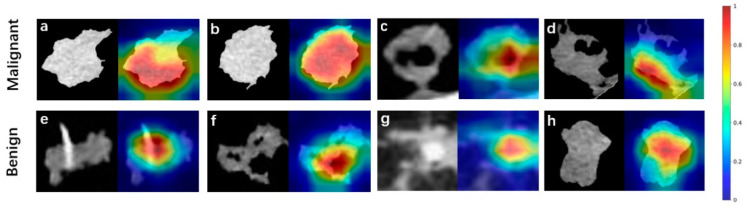
Feature maps of eight examples from validation set. Pulmonary nodules of the upper row (**a**–**d**) and lower row (**e**–**h**) are malignant and benign, respectively. Pictures (**a**,**b**,**e**,**f**) are correctly classified as malignant or benign nodules, whereas (**c**,**d**,**g**,**h**) are not.

Figure 4 shows feature maps of eight examples from the validation set. The active areas (areas of bright colors) represent the focus areas of the model. The feature maps demonstrated that they were consistent with human observations, the region of the whole lesion, or a specific area helpful in diagnoses.

**Figure 5:**
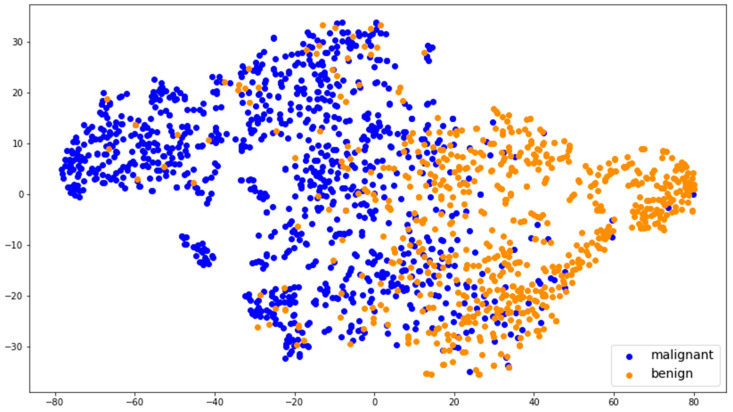
Visualization of the effectiveness of the learned features.

The *t*-SNE visualization demonstrated how the model clustered malignant and benign nodules by using learned features (Figure 5). Nodules with similar features are close to one another, and those with dissimilar features are not. The colored point clusters were well separated, indicating that effective features were captured by the model.

## 4. Discussion

Accurate estimation of the benign and malignant lung nodules found in CT is essential for the early diagnosis of lung cancer, and it is still a challenging task for radiologists. In this study, we used CT images, morphological features, and clinical information with pulmonary nodules in four hospitals to develop and verify the deep-learning and machine-learning classification models to differentiate between benign and malignant pulmonary nodules. Our study found that the CBAM-ResNet model, which utilized images, morphological features, and clinical information, demonstrated good differentiating performance and outperformed the radiomic model.

In recent years, CNNs have been applied in many studies for medical image classification, involving different diseases [23,33,34]. Many studies [11,12,13,14] mentioned above have confirmed that the deep-learning network can achieve an accurate classification result. CNN models could automatically detect the image features and summarize some useful points by updating the massive weight parameters [8]. ResNet is improved on the basis of using a visual geometry group (VGG) network, which has fewer filters and lower computational complexity. ResNet makes it easier to optimize the network by adding a short-circuit connection structure, and it can improve accuracy by increasing the depth. CBAM is a simple and effective attention module for feedforward convolutional neural networks. Given an intermediate feature map, the CBAM module sequentially infers the attention map along two dimensions (channel and space) and then multiplies the attention map with the input feature map for adaptive feature optimization [35]. Our results showed that combining CBAM with ResNet could achieve a good classification result (AUC = 0.940, accuracy = 0.867) that takes only images as inputs. It implied that CNNs could to some extent learn useful features directly from the images.

The morphological features of the pulmonary nodule, involving halo sign, lobulation sign, speculation sign, ground-glass opacity sign, and others, could be measured by radiologists, depending on the CT images. Among the morphological features, the incidences of six signs (diameter, halo, lobulation, vacuole, spiculation, and pleural retraction) in the malignant nodules were higher than those in the benign group, while the incidences of calcification and cavity were lower. These features are critical for classification in clinical practice and are validated to be significantly effective in the prediction of lung cancer from the clinical perspective [19]. Incorporating morphological features can achieve high accuracy for the pulmonary nodule’s classification in both the deep-learning and machine-learning methods. The image sign information can provide information other than the simple nodule image and can help doctors make preliminary diagnoses and improve interpretability [36]. Previous studies have shown that older age and a heavy smoking history are associated with an increased risk of developing lung cancer, while the association between gender and lung cancer risk is complex and not fully understood [37]. In our study, we found that age and smoking showed statistically significant differences between the malignant lung nodule group and the benign lung nodule group, while gender did not show any statistically significant differences. These results suggest that incorporating age and smoking information into our deep-learning model may improve its performance for classifying lung nodules. Adding these two factors to the model increased its accuracy and its AUC to 0.898 and 0.957, respectively.

In comparison with other recently published research, we found that Dhara et al. proposed an SVM-based lung nodule classification method that combined shape and texture features for classification of pulmonary nodules in lung CT images, with a sensitivity of 0.897 and a specificity of 0.864. However, their method requires manual intervention and evaluates only a subset of the LIDC-IDRI data set [38]. Liu et al. proposed 2D CNN-based methods that utilize only the central layer’s characteristic information of the nodule, resulting in less than 70% sensitivity and suboptimal performance [39]. On the other hand, both Liu et al. and Shen et al. proposed a 3D CNN-based classification method for benign and malignant lung nodules that made better use of the spatial information of nodules and achieved improved performance [40,41]. Additionally, Liu et al. proposed a multimodel integrated learning architecture for the classification of suspicious lung nodules and malignant tumors, achieving good classification results with an accuracy of 0.906 and an AUC of 0.939 [42]. Our research achieved better classification performance by simultaneously inputting images, morphological information, and clinical information, which allowed for a more comprehensive analysis of lung nodules.

There are some advantages of our study. First, a novel deep-learning model (CBAM-ResNet network) based on CT images, morphological features, and clinical information with high performance was constructed and developed and then compared with the traditional machine-learning methods. Second, the research subjects in our study have definite diagnoses, and the benign and malignant results were obtained via pathological diagnosis, so we can obtain more-accurate models. Lastly, the imaging data were collected from a multicenter study, so they can reduce the deviation caused by having different patients in different hospitals.

Nevertheless, there are also some limitations in our study. First, this study is a cross-sectional study, and its data were collected retrospectively. Follow-ups on benign nodules for 2 years or more can lead to more-accurate results. Second, we did not compare the model with the diagnoses of radiologists, which ultimately did not indicate the possibility of a difference between the classification model discussed in the design of this study and the diagnostic ability of radiologists. Lastly, one limitation of our deep-learning algorithm is its reliance on large amounts of accurately labeled data, which is compounded by the additional complexity of incorporating imaging and clinical information into our model, making data collection more challenging.

In conclusion, the deep-learning model CBAM-ResNet, which trained on participants with lung nodules from multiple centers, showed excellent performance in the identification of malignant and benign lung nodules. Incorporating morphological and clinical information can further enhance the model’s classification performance. The developed model could aid clinicians in accurately diagnosing pulmonary nodules in clinical practice.

## Figures and Tables

**Table 1:** The checklist of subjects and images.

Source	Subjects (*n*, %)	Images (*n*, %)
Beijing Chest Hospital	183 (18.8)	3065 (37.2)
Beijing Friendship Hospital	174 (17.9)	1454 (17.6)
Beijing Cancer Hospital	394 (40.5)	2312 (28.1)
Xuanwu Hospital	221 (22.7)	1410 (17.1)
Total	972 (100.0)	8241 (100.0)

**Table 2:** Demographic characteristics of the patients in the training and testing sets.

Characteristic	Training Set (*n* = 6594)	Testing Set (*n* = 1647)	*p*-Value
Age (years, mean ± SD)	61.5 ± 11.6	61.3 ± 11.8	0.451 ^a^
Sex (*n*, %)			0.391 ^b^
Male	4171 (63.3)	1023 (62.1)	
Female	2423 (36.7)	624 (37.9)	
Type (*n*, %)			0.966 ^b^
Malignant	4103 (62.2)	1027 (62.3)	
Benign	1491 (37.8)	620 (37.7)	

^a^ *t*-test was used for the distribution difference of continuous variables. ^b^ Chi-square test was used for the distribution difference of categorical variables.

**Table 3:** Morphological feature distribution of images.

Morphological Feature	Benign (*n* = 3111)	Malignant (*n* = 5130)	*p*-Value
Diameter (cm, mean ± SD)	2.582 ± 1.30	3.135 ± 1.52	<0.001 ^a^
Halo (yes, %)	47 (3.4)	248 (6.2)	<0.001 ^b^
Lobulation (yes, %)	477 (34.8)	3073 (73.6)	<0.001 ^b^
Vacuole (yes, %)	46 (2.7)	575 (14.6)	<0.001 ^b^
Spiculation (yes, %)	245 (16.9)	1802 (45.9)	<0.001 ^b^
Calcification (yes, %)	316 (22.0)	324 (5.3)	<0.001 ^b^
Cavity (yes, %)	115 (8.6)	231 (6.0)	<0.001 ^b^
Pleural retraction (yes, %)	181 (14.1)	1871 (47.0)	<0.001 ^b^

^a^ *t*-test was used for the distribution difference of continuous variables. ^b^ Chi-square test was used for the distribution difference of categorical variables.

**Table 4:** Classification results of four models in the test set.

Model	AUC (95% CI)	Accuracy	Sensitivity	Specificity
CBAM-ResNet1	0.940 (0.927–0.953)	0.867	0.931	0.849
CBAM-ResNet2	0.955 (0.939–0.972)	0.896	0.968	0.863
CBAM-ResNet3	0.957 (0.942–0.975)	0.898	0.971	0.864
NSDTCT-SVM1	0.692 (0.676–0.708)	0.691	0.748	0.691
NSDTCT-SVM2	0.791 (0.772–0.811)	0.765	0.752	0.776
NSDTCT-SVM3	0.807 (0.795–0.819)	0.779	0.763	0.782

CBAM-ResNet1 is a CBAM-ResNet framework that uses only images as inputs; CBAM-ResNet2 adds morphological features to CBAM-ResNet. NSDTCT-SVM1 uses only texture features after a LASSO regression; NSDTCT-SVM2 uses texture features and eight morphological features. CBAM-ResNet3 and NSDTCT-SVM3 add age and smoking on the basis of results from CBAM-ResNet2 and NSDTCT-SVM2, respectively.

## Data Availability

The data for this study are available from the authors upon reasonable request.

## References

[1] Sung H., Ferlay J., Siegel R.L., Laversanne M., Soerjomataram I., Jemal A., Bray F. (2021). Global Cancer Statistics 2020: GLOBOCAN Estimates of Incidence and Mortality Worldwide for 36 Cancers in 185 Countries. CA Cancer J. Clin..

[2] Lu H., Jiang Z. (2018). Advances in antibody therapeutics targeting small-cell lung cancer. Adv. Clin. Exp. Med..

[3] Thawani R., McLane M., Beig N., Ghose S., Prasanna P., Velcheti V., Madabhushi A. (2018). Radiomics and radiogenomics in lung cancer: A review for the clinician. Lung Cancer.

[4] Siegel R.L., Miller K.D., Jemal A. (2020). Cancer statistics, 2020. CA Cancer J. Clin..

[5] Yu W., Tang C., Hobbs B.P., Li X., Koay E.J., Wistuba I.I., Sepesi B., Behrens C., Rodriguez Canales J., Parra Cuentas E.R. (2018). Development and Validation of a Predictive Radiomics Model for Clinical Outcomes in Stage I Non-small Cell Lung Cancer. Int. J. Radiat. Oncol. Biol. Phys..

[6] Li W., Wang X., Zhang Y., Li X., Li Q., Ye Z. (2018). Radiomic analysis of pulmonary ground-glass opacity nodules for distinction of preinvasive lesions, invasive pulmonary adenocarcinoma and minimally invasive adenocarcinoma based on quantitative texture analysis of CT. Chin. J. Cancer Res..

[7] Lim K.P., Marshall H., Tammemägi M., Brims F., McWilliams A., Stone E., Manser R., Canfell K., Weber M., Connelly L. (2020). Protocol and Rationale for the International Lung Screening Trial. Ann. Am. Thorac. Soc..

[8] Xu K., Feng D., Mi H. (2017). Deep Convolutional Neural Network-Based Early Automated Detection of Diabetic Retinopathy Using Fundus Image. Molecules.

[9] Jiang S., Li H., Jin Z. (2021). A Visually Interpretable Deep Learning Framework for Histopathological Image-Based Skin Cancer Diagnosis. IEEE J. Biomed. Health Inform..

[10] Cetinoglu Y.K., Koska I.O., Uluc M.E., Gelal M.F. (2021). Detection and vascular territorial classification of stroke on diffusion-weighted MRI by deep learning. Eur. J. Radiol..

[11] Marentakis P., Karaiskos P., Kouloulias V., Kelekis N., Argentos S., Oikonomopoulos N., Loukas C. (2021). Lung cancer histology classification from CT images based on radiomics and deep learning models. Med. Biol. Eng. Comput..

[12] Xu Y., Hosny A., Zeleznik R., Parmar C., Coroller T., Franco I., Mak R.H., Aerts H.J.W.L. (2019). Deep Learning Predicts Lung Cancer Treatment Response from Serial Medical Imaging. Clin. Cancer Res..

[13] Liu S., Xie Y., Jirapatnakul A., Reeves A.P. (2017). Pulmonary nodule classification in lung cancer screening with three-dimensional convolutional neural networks. J. Med. Imaging.

[14] Yang Y., Feng X., Chi W., Li Z., Duan W., Liu H., Liang W., Wang W., Chen P., He J. (2018). Deep learning aided decision support for pulmonary nodules diagnosing: A review. J. Thorac. Dis..

[15] Gillies R.J., Kinahan P.E., Hricak H. (2016). Radiomics: Images Are More than Pictures, They Are Data. Radiology.

[16] Lambin P., Rios-Velazquez E., Leijenaar R., Carvalho S., van Stiphout R.G.P.M., Granton P., Zegers C.M.L., Gillies R., Boellard R., Dekker A. (2012). Radiomics: Extracting more information from medical images using advanced feature analysis. Eur. J. Cancer.

[17] Bian Y., Jiang H., Ma C., Wang L., Zheng J., Jin G., Lu J. (2020). CT-Based Radiomics Score for Distinguishing Between Grade 1 and Grade 2 Nonfunctioning Pancreatic Neuroendocrine Tumors. AJR Am. J. Roentgenol..

[18] Li J., Zhang C., Wei J., Zheng P., Zhang H., Xie Y., Bai J., Zhu Z., Zhou K., Liang X. (2020). Intratumoral and Peritumoral Radiomics of Contrast-Enhanced CT for Prediction of Disease-Free Survival and Chemotherapy Response in Stage II/III Gastric Cancer. Front. Oncol..

[19] Shen S., Han S.X., Aberle D.R., Bui A.A., Hsu W. (2019). An Interpretable Deep Hierarchical Semantic Convolutional Neural Network for Lung Nodule Malignancy Classification. Expert Syst. Appl..

[20] Lei Y., Tian Y., Shan H., Zhang J., Wang G., Kalra M.K. (2020). Shape and margin-aware lung nodule classification in low-dose CT images via soft activation mapping. Med. Image Anal..

[21] Khan M.A., Rajinikanth V., Satapathy S.C., Taniar D., Mohanty J.R., Tariq U., Damaševičius R. (2021). VGG19 Network Assisted Joint Segmentation and Classification of Lung Nodules in CT Images. Diagnostics.

[22] Cai Z., Vasconcelos N. (2021). Cascade R-CNN: High Quality Object Detection and Instance Segmentation. IEEE Trans. Pattern Anal. Mach. Intell..

[23] Huang Q., Li W., Zhang B., Li Q., Tao R., Lovell N.H. (2020). Blood Cell Classification Based on Hyperspectral Imaging With Modulated Gabor and CNN. IEEE J. Biomed. Health Inform..

[24] Balagourouchetty L., Pragatheeswaran J.K., Pottakkat B., Ramkumar G. (2020). GoogLeNet-Based Ensemble FCNet Classifier for Focal Liver Lesion Diagnosis. IEEE J. Biomed. Health Inform..

[25] Geng L., Zhang S., Tong J., Xiao Z. (2019). Lung segmentation method with dilated convolution based on VGG-16 network. Comput. Assist. Surg..

[26] He F., Liu T., Tao D. (2020). Why ResNet Works? Residuals Generalize. IEEE Trans. Neural Netw. Learn. Syst..

[27] Tao Z., Bingqiang H., Huiling L., Zaoli Y., Hongbin S. (2020). NSCR-Based DenseNet for Lung Tumor Recognition Using Chest CT Image. Biomed. Res. Int..

[28] Li L., Yang Y., Zhang Q., Wang J., Jiang J., Neuroimaging Initiative A.s.D. (2021). Use of Deep-Learning Genomics to Discriminate Healthy Individuals from Those with Alzheimer’s Disease or Mild Cognitive Impairment. Behav. Neurol..

[29] Hu J., Shen L., Albanie S., Sun G., Wu E. (2020). Squeeze-and-Excitation Networks. IEEE Trans. Pattern Anal. Mach. Intell..

[30] Feng W., Halm-Lutterodt N.V., Tang H., Mecum A., Mesregah M.K., Ma Y., Li H., Zhang F., Wu Z., Yao E. (2020). Automated MRI-Based Deep Learning Model for Detection of Alzheimer’s Disease Process. Int. J. Neural Syst..

[31] Wu P., Sun X., Zhao Z., Wang H., Pan S., Schuller B. (2020). Classification of Lung Nodules Based on Deep Residual Networks and Migration Learning. Comput. Intell. Neurosci..

[32] Kavitha M.S., Shanthini J., Sabitha R. (2019). ECM-CSD: An Efficient Classification Model for Cancer Stage Diagnosis in CT Lung Images Using FCM and SVM Techniques. J. Med. Syst..

[33] Basaia S., Agosta F., Wagner L., Canu E., Magnani G., Santangelo R., Filippi M. (2019). Automated classification of Alzheimer’s disease and mild cognitive impairment using a single MRI and deep neural networks. Neuroimage Clin..

[34] Girard F., Kavalec C., Cheriet F. (2019). Joint segmentation and classification of retinal arteries/veins from fundus images. Artif. Intell. Med..

[35] Xiao Y., Yin H., Wang S.-H., Zhang Y.-D. (2021). TReC: Transferred ResNet and CBAM for Detecting Brain Diseases. Front. Neuroinform..

[36] Vlahos I., Stefanidis K., Sheard S., Nair A., Sayer C., Moser J. (2018). Lung cancer screening: Nodule identification and characterization. Transl. Lung Cancer Res..

[37] Li L., Shao M., He X., Ren S., Tian T. (2021). Risk of lung cancer due to external environmental factor and epidemiological data analysis. Math. Biosci. Eng..

[38] Dhara A.K., Mukhopadhyay S., Dutta A., Garg M., Khandelwal N. (2016). A Combination of Shape and Texture Features for Classification of Pulmonary Nodules in Lung CT Images. J. Digit. Imaging.

[39] Liu K., Kang G. (2017). Multiview convolutional neural networks for lung nodule classification. Int. J. Imaging Syst. Technol..

[40] Liu Y., Hao P., Peng Z., Xu X., Wei C.J.I.A. (2018). Dense Convolutional Binary-Tree Networks for Lung Nodule Classification. IEEE Access.

[41] Shen W., Zhou M., Yang F., Yu D., Dong D., Yang C., Zang Y., Tian J. (2017). Multi-crop Convolutional Neural Networks for lung nodule malignancy suspiciousness classification. Pattern Recognit..

[42] Liu H., Cao H., Song E., Ma G., Xu X., Jin R., Liu C., Hung C.-C. (2020). Multi-model Ensemble Learning Architecture Based on 3D CNN for Lung Nodule Malignancy Suspiciousness Classification. J. Digit. Imaging.

